# XRCC3 Thr241Met Is Associated with Response to Platinum-Based Chemotherapy but Not Survival in Advanced Non-Small Cell Lung Cancer

**DOI:** 10.1371/journal.pone.0077005

**Published:** 2013-10-08

**Authors:** Mantang Qiu, Lei Xu, Xin Yang, Xiangxiang Ding, Jingwen Hu, Feng Jiang, Lin Xu, Rong Yin

**Affiliations:** 1 Department of Thoracic Surgery, Nanjing Medical University Affiliated Cancer Institute of Jiangsu Province, Nanjing, China; 2 The Fourth Clinical College of Nanjing Medical University, Nanjing, China; 3 The First Clinical College of Nanjing Medical University, Nanjing, China; University of Torino, Italy

## Abstract

**Background:**

A lot of studies have investigated the correlation between x-ray repair cross-complementing group 3 (XRCC3) Thr241Met polymorphism and clinical outcomes in non-small cell cancer (NSCLC), while the conclusion is still conflicting.

**Materials and Methods:**

We conducted this meta-analysis to evaluate the predictive value of XRCC3 Thr241Met polymorphism on response and overall survival of patients with NSCLC. Pooled odds ratios (ORs) and hazard ratios (HRs) and corresponding 95% confidence intervals (95% CIs) were used to estimate the association strength.

**Results:**

A total of 14 eligible studies with 2828 patients were identified according to our inclusion criteria. Meta-analysis results showed that carriers of the variant 241Met allele were significantly associated with good response, compared with those harboring the wild 241Thr allele (Met vs. Thr, OR = 1.453, 95% CI: 1.116–1.892, P_heterogeneity_ = 0.968 and ThrMet+MetMet vs. ThrThr, OR = 1.476, 95% CI: 1.087–2.004, P_heterogeneity_ = 0.696). This significant association was observed in Caucasian population but not in Asian population. On the other hand, there was no significant association of XRCC3 Thr241Met polymorphism with survival (ThrMet+MetMet vs. ThrThr, HR = 1.082, 95% CI: 0.929–1.261, P_heterogeneity_ = 0.564), and there was no difference between Asian and Caucasian population.

**Conclusions:**

These findings suggest a predictive role of XRCC3 Thr241Met polymorphism on response to platinum-based chemotherapy in patients with advanced NSCLC. Additionally, we first report that the XRCC3 Thr241Met polymorphism is associated with response to platinum-based chemotherapy and highlights the prognostic value of the XRCC3 Thr241Met polymorphism.

## Introduction

Non-small cell lung cancer (NSCLC) accounts for about 80% of primary lung cancers, most of which were diagnosed at the advanced stage [1]. Chemotherapy is the main treatment of choice for advanced NSCLC [2], [3]. Among various cytotoxic drugs, platinum is the most extensively used chemotherapeutic agent in lung cancer treatment and platinum-based doublet chemotherapy has been recommended by lots of clinical guidelines. Despite the improvement made to chemotherapy in the last 2 decades, the current response rate to platinum-based regimen is about 19% in patients with advanced NSCLC and the median survival is only 7–9 months. In addition, the response to platinum-based chemotherapy varies greatly among individuals[4], [5].

A lot of clinical studies have suggested that genetic factors can influence treatment efficacy of lung cancer and are correlated with prognosis of patients [6]–[8]. Among these genetic factors, single nucleotide polymorphisms (SNPs) in the DNA repair pathway have been mostly investigated. X-ray repair cross-complementing group 3 (XRCC3) protein, a member of the double-strand break (DSB) repair pathway, plays a direct role in homologous recombination that is important for the integrity of chromosome and repair of damaged DNA. It has been suggested that the functional SNP in codon 241 (Thr to Met, rs861539 C>T) of XRCC3 is associated with risk of lung cancer [9], [10] and survival of NSCLC [11], [12]. For example, in an observational study of 358 NSCLC patients, Chen X and colleagues [12] found that carriers of the variant 241Met allele were correlated with a longer survival in the patients treated with platinum-gemcitabine regimen. While de las Peñas R et al showed that patients with MetMet and ThrMet genotypes were associated with a longer survival compared with those harboring ThrThr genotype[11]. In addition, other studies found no association of XRCC3 Thr241Met polymorphism with survival [13]–[15]. Thus, the conclusion is conflicting and a systematic review of published evidence is needed.

Therefore, this present meta-analysis was carried out to evaluate the predictive value of XRCC3 Thr241 polymorphism by analyzing the relationship between XRCC3 Thr241Met polymorphism and response to platinum-based chemotherapy and survival of NSCLC.

## Materials and Methods

### Data sources and searching strategy

This meta-analysis was conducted and reported in accordance with the PRISMA guidelines (Checklist S1. PRISMA Checklists) [16]. A comprehensive search was performed in online databases of PubMed, EMBASE and China National Knowledge Infrastructure (CNKI) to identify potentially relevant studies. The searching strategy consisted of combinations of medical subheadings and key words such as “lung neoplasms” or “lung cancer” and “x-ray repair cross-complementing group 3” or “XRCC3” and “polymorphisms, single nucleotide” or “polymorphism”. Other alternative spellings were also considered. The last search was performed in May 2013. References lists of related review articles and original studies were manually searched to identify studies missed by the database search.

### Study identification and inclusion criteria

Records identified from databases were primarily screened by titles and abstracts, and then full-text articles were retrieved to further assess the eligibility. Studies met the following criteria were included: 1) NSCLC patients; 2) investigating the relationship between XRCC3 Thr241Met polymorphism and response to chemotherapy or survival; 3) for response to chemotherapy, the regimen was restricted to platinum-based chemotherapy; 4) for survival, there was no limitation on treatment methods; 5) available data for quantitative synthesis, namely genotype distribution data for response or hazard ratio (HR) and 95% confidence intervals (CIs) for survival. Conference abstracts were excluded and only full-text published articles were included. Studies without available data were excluded. All searching records were screened by two authors (Qiu and Yang), with discrepancies resolved by discussion with another author (Yin).

### Outcomes definition

Response to platinum-based chemotherapy and overall survival were the primary outcomes in this meta-analysis. Response to chemotherapy was assessed with RECIST (response evaluation criteria in solid tumors) criteria, namely, “good response” was defined as “complete response + partial response” and “poor response” was “stable disease + progressive disease”. Data of overall survival (HR and 95% CIs) were extracted from studies directly according to studies' own definition.

### Data extraction

Data of eligible studies were extracted by two authors (Qiu and Xu) independently in duplicate with a pre-designed data collection form. The two authors reached consensus on each item. The following data was collected, name of first author, year of publication, country, ethnicity, treatment (chemotherapy regimens), number of patients, TNM stages, age, percentage of male, SNPs investigated, genotype distribution data among responders and non-responders and HR and corresponding 95% CIs of OS. For OS, we collected HR and CIs of each comparison. Ethnicity descents were simply categorized as Asian or Caucasian. In three studies [11], [12], [17], the HR and 95% CIs was not presented but Kaplan-Meier curves were available, thus, the HRs and 95% CIs were estimated from Kaplan-Meier curves using the method introduced by Tierney [18].

### Statistical analysis

Pooled odds ratio (OR) and corresponding 95% CIs were calculated to estimate the association strength of XRCC3 Thr241Met polymorphisms with response to platinum-based chemotherapy. The 95% CIs were utilized for statistical significance test and a 95% CI without 1 suggested significant difference. For response, a total of 5 genetic comparison models were calculated (A: allele comparison, Met vs. Thr; B: heterozygote comparison, ThrMet vs. ThrThr; C: homozygote comparison, MetMet vs. ThrThr; D: dominant model, ThrMet+MetMet vs. ThrThr; E: recessive model, MetMet vs. ThrThr+ ThrMet; A, variant allele; a, wild allele; the 241Met was considered as the variant allele). The genotype distribution data was directly used to estimate the pooled ORs and 95% CIs. For OS, HRs and CIs retrieved from each study were calculated to estimate the pooled HRs and 95% CIs. Also, the 95% CIs pooled HRs were used for statistical test. Pooled HRs for homozygote comparison, heterozygote comparison and dominant model were calculated.

Between studies heterogeneity was measured by chi-square based Q test, and p<0.10 indicated the existence of significant heterogeneity [19]. The fixed-effects model and random-effects model were utilized to pool data from eligible studies. The fixed-effects model was used in the absence of significant heterogeneity; otherwise, the random-effects model was applied. Sub-group analyses according to ethnicities. Begg's funnel plot and Egger's linear regression test were conducted to detect publication bias, and a p<0.05 was considered significant [20].

All statistical analyses were carried out with STATA software (version 11.0, StataCorp, College Station, Texas USA). All p values are two-side.

## Results

**Figure 1:**
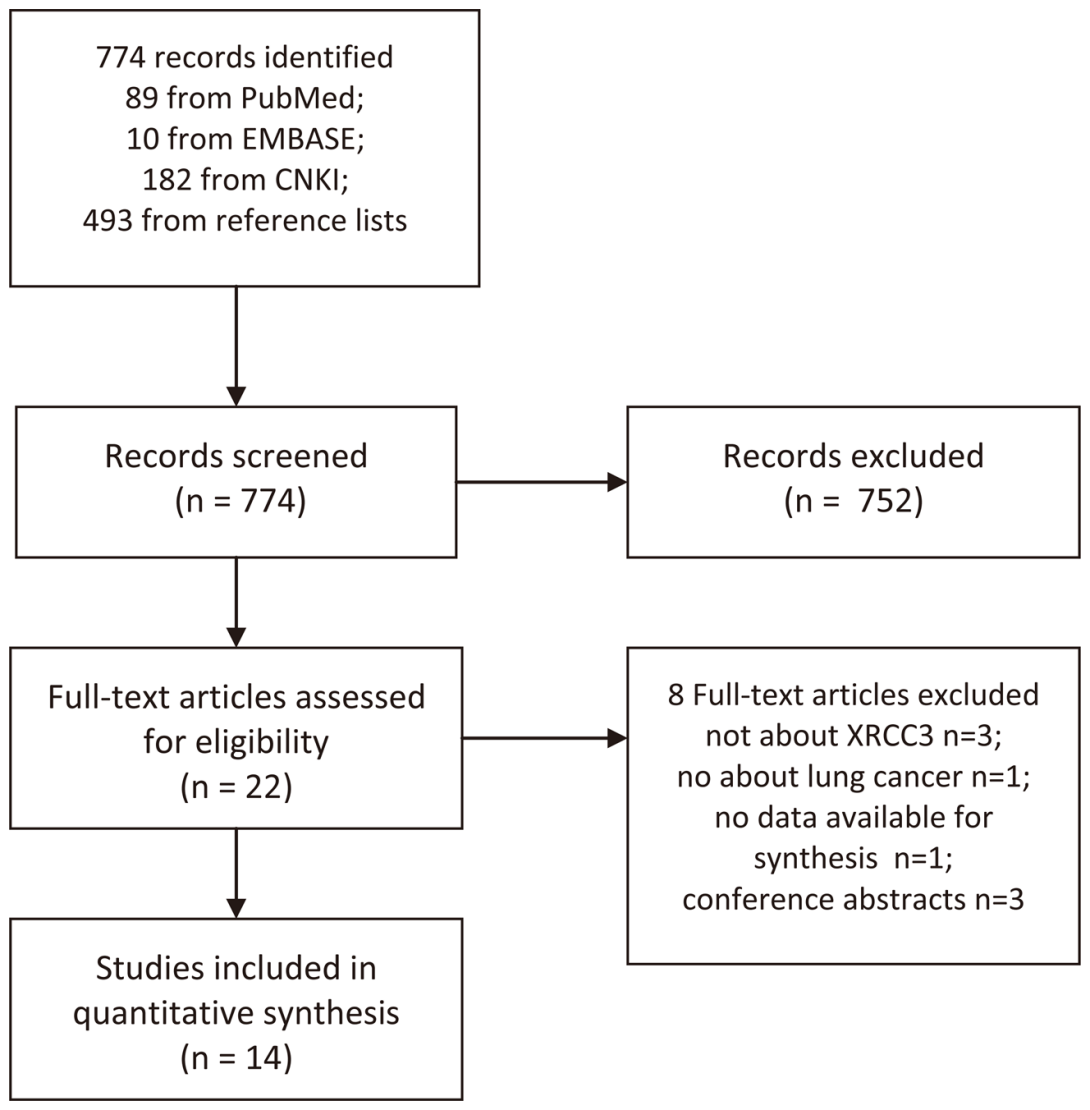
Flow Diagram.

After primary screening, 774 potentially relevant full-text articles were identified, of which 14 studies met the inclusion criteria[11]–[15], [17], [21]–[28]. The process of study identification is shown in Figure 1. As a result, a total of 2828 patients with NSCLC were included in this meta-analysis. Most of the patients were at advanced stage (IIIB-IV). The treatment methods included platinum-based chemotherapy, surgery and radiotherapy+chemotherapy. Notably, in the study reported by Liao WY [23], a small proportion of patients received bevacizumab (13 of 62). The baseline characteristics of eligible studies are shown in Table 1.

**Table 1:** Baseline Characteristics of Eligible Studies.

Author	Year	Country	Ethnicity	Chemotherapy	TNM Stage	Num	Age	Male
Butkiewicz D	2012	Poland	Caucasian	radiotherapy+chemotherapy	III-IV 90%	250	<62, 48%	65%
Chen X	2011	China	Asian	cisplatin/carboplatin+gemcitabine/vinorelbine/paclitaxel/docetaxel	IV66.8%/III B 33.2%	355	60(32–78)	69.9%
de las Peñas R	2005	Italy	Caucasian	cisplatin+gemcitabine	III B 17%/IV83%	135	62 (31–81)	92.6%
Joerger M	2011	Netherlands	Mixed	gemcitabine+cisplatin/carboplatin	IV69%/IIIB 31%	137	59.7(37–79)	56%
Ke HG	2012	China	Asian	cisplatin/gemcitabine/docetaxel/vinorelbine/paclitaxel	/IV39.1%/III 26.6%	460	59.5±3.5	72.6%
Liao WY †	2012	China	Asian	gemcitabine/bevacizumab+cisplatin/carboplatin/oxaliplatin	IV84%/III B 16%	62	57(36–78)	56.5%
Ludovini V	2011	Italy	Caucasian	cisplatin+gemcitabine/vinorelbine/taxol/gemcitabine alone	IV76%/III B 24%	192	63(25–81)	74.0%
Osawa K	2012	Japan	Asian	NA	III-IV 20.2%	99	66.3±9.3	65.7%
Provencio M	2012	Spain	Caucasian	cisplatin+vinorelbine	IV83%/III B 17%	180	62(39–78)	87.2%
Ren S	2011	China	Asian	cisplatin+gemcitabine/docetaxel/vinorelbine/paclitaxel	IV67.4%/III B 32.6%	340	60(30–78)	68.2%
Ren SX	2009	Chian	Asian	cisplatin/carboplatin+gemcitabine/vinorelbine/paclitaxel	IV69.9%/IIIB30.1%	130	61(30–78)	56.9%
Xu C	2011	China	Asian	cisplatin+gemcitabine/docetaxel/vinorelbine/paclitaxel	advanced	130	62(28–83)	69.2%
Yin M	2011	China	Asian	radiotherapy+chemotherapy	III-IV 82.5%	228	63(35–88)	54.8%
Zhou C	2010	China	Asian	cisplatin+gemcitabine/vinorelbine/paclitaxel	IV69.2%/III B 30.8%	130	61(30–78)	56.9%

Age is presented as median and range or mean±standard deviation; † data was extracted from training set.

### XRCC3 Thr241Met polymorphism and response to platinum-based chemotherapy

**Figure 2:**
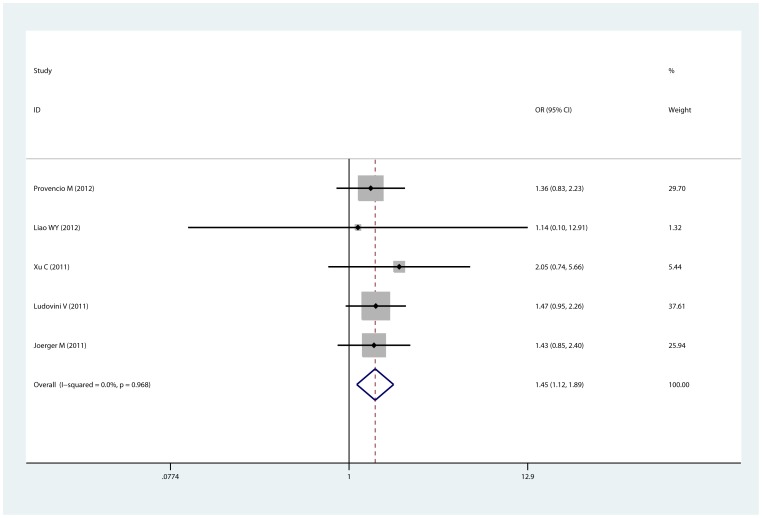
Forest plot of the comparison of Mer vs. Thr for response to platinum-based chemotherapy. OR = 1.453, 95% CI: 1.116–1.892, p = 0.968 for heterogeneity.

Eight studies [12], [14], [17], [22]–[25], [28] of 1289 patients were eligible for the analysis, including 5 Asian studies and 2 Caucasian studies. Doublet regimens were used in most studies. Cisplatin and carboplatin were the most common platinum agents. As shown in Table 2, compared with the 241Thr allele, the XRCC3 241Met allele was significantly associated with good response to platinum-based chemotherapy in overall analysis. This significant association was observed in allele comparison (Met vs. Thr, OR = 1.453, 95% CI: 1.116–1.892, p = 0.968 for heterogeneity; Figure 2), homozygote comparison, heterozygote comparison and dominant model (ThrMet+MetMet vs. ThrThr, OR = 1.476, 95% CI: 1.087–2.004, p = 0.696 for heterogeneity). In subgroup analysis by ethnicity, the XRCC3 241Met allele was significantly correlated with good response in Caucasian population (Met vs. Thr, OR = 1.421, 95% CI: 1.028–1.964, p = 0.868 for heterogeneity), but the association was not observed in Asian population. No evidence of publication bias was found (p = 1 for Begg's test and p = 0.934 for Egger's test in dominant model, in which all 8 studies were included; Figure S1).

**Table 2:** The XRCC3 Thr241Met polymorphism and response to platinum-based chemotherapy.

		Allele Comparison			Homozygote Comparison			Heterozygote Comparison			Recessive Model			Dominant Model	
	Num	OR(95% CI)	P	Num	OR(95% CI)	P	Num	OR(95% CI)	P	Num	OR(95% CI)	P	Num	OR(95% CI)	P
Overall	5	1.453 (1.116–1.892)*	0.968	3	1.983 (1.092–3.599)*	0.868	5	1.744 (1.169–2.601)*	0.527	3	1.390 (0.839–2.303)	0.343	8	1.476 (1.087–2.004)*	0.696
Ethnicity															
Asian	2	1.871 (0.738–4.748)	0.66	NA	NA	NA	2	1.953 (0.745–5.120)	0.638	NA	NA	NA	5	1.179 (0.745–1.866)	0.776
Caucasian	2	1.421 (1.028–1.964)*	0.828	2	2.148 (1.073–4.298)*	0.78	2	1.349 (0.807–2.254)	0.875	2	1.751 (0.967–3.172)	0.695	2	1.509 (0.928–2.455)	0.803
Mixed	1	1.428 (0.849–2.403)	NA	1	1.569 (0.483–5.098)	NA	1	3.243 (1.349–7.795)*	NA	1	0.755 (0.275–2.075)	NA	1	2.716 (1.169–6.309)*	NA

Allele Comparison: Met vs. Thr; Heterozygote Comparison: ThrMet vs. ThrThr; Homozygote Comparison: MetMet vs. ThrThr; Recessive Model: MetMet vs. ThrThr+ThrMet; Dominant Model: ThrMet+MetMet vs. ThrThr; P: p value for heterogeneity; Num: number of studies analyzed; NA: not available; * significant difference.

### XRCC3 Thr241Met polymorphism and overall survival

**Figure 3:**
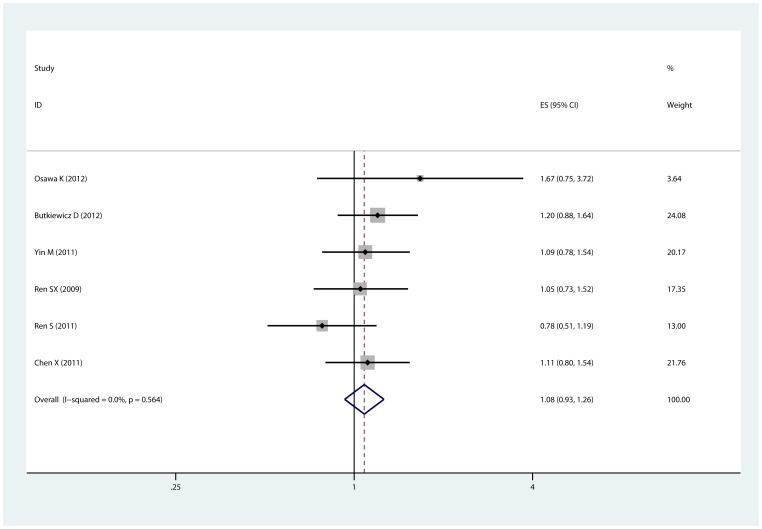
Forest plot of the comparison of ThrMet+MetMet vs. ThrThr for overall survival. HR = 1.082, 95% CI: 0.929–1.261, p = 0.564 for heterogeneity.

For overall survival, we did not restrict the treatment methods. Thus, 10 studies [11]–[15], [17], [21], [23], [26], [27] with 2196 patients were eligible for evaluating the relationship between XRCC3 Thr241Met polymorphism and survival of NSCLC receiving surgery, platinum-based chemotherapy and radiotherapy+chmotherapy. In overall analysis, as shown in Table 3, 3 comparison models were conducted and no significant association of Thr241Met polymorphism with survival was observed in any of the 3 comparisons (ThrMet+MetMet vs. ThrThr, HR = 1.082, 95% CI: 0.929–1.261, p = 0.564 for heterogeneity, Figure 3; ThrMet vs. ThrThr, HR =  1.220, 95% CI: 0.957–1.555, p = 0.056 for heterogeneity, Figure S2). However, in heterozygote comparison, when pooling by fixed-effects model, the ThrMet genotype was associated with significantly poor survival (ThrMet vs. ThrThr, HR = 1.357, 95% CI: 1.211–1.521, Figure S3). Stratified analysis by ethnicity showed no association neither in Asian nor in Caucasian population. We further analyzed the role of XRCC3 Thr241Met polymorphism in different treatment. Subgroup analysis revealed that the Thr241Met polymorphism was not correlated with survival in NSCLC patients receiving surgery, chemotherapy or radiotherapy+chemotherapy. Similarly, no evidence of publication bias was detected (p = 0.806 for Begg's test and p = 0.722 for Egger's test in dominant model).

**Table 3:** The association of XRCC3 Thr241Met polymorphism and survival of NSCLC.

		Heterozygote Comparison			Homozygote Comparison			Dominant Model	
	Num	HR(95% CI)	P	Num	HR(95% CI)	P	Num	HR(95% CI)	P
Overall	6	1.220 (0.957–1.555)	0.056	4	0.891 (0.752–1.056)	0.222	6	1.082 (0.929–1.261)	0.564
Ethnicity									
Asian	4	1.077 (0.798–1.453)	0.289	2	0.830 (0.593–1.162)	0.485	5	1.047 (0.879–1.248)	0.503
Caucasian	1	1.500 (1.309–1.719)*	NA	1	0.850 (0.689–1.049)	NA	1	1.200 (0.879–1.638)	NA
Mixed	1	1.120 (0.737–1.703)	NA	1	1.540 (0.871–2.723)	NA	NA	NA	NA
Treatment									
Pt-Chemo	4	1.155 (0.803–1.662)	0.017	3	0.887 (0.737–1.068)	0.112	3	0.998 (0.808–1.233)	0.411
Surgery	1	1.670 (0.750–3.719)	NA	NA	NA	NA	1	1.670 (0.750–3.719)	NA
Radio+Chemo	1	1.220 (0.840–1.771)	NA	1	0.910 (0.596–1.390)	NA	2	1.149 (0.913–1.445)	0.683

Heterozygote Comparison: ThrMet vs. ThrThr; Homozygote Comparison: MetMet vs. ThrThr; Dominant Model: ThrMet+MetMet vs. ThrThr; P: p value for heterogeneity; Pt-chemo: platinum-based chemotherapy; Num: number of studies analyzed; Radio+Chemo: radiotherapy + chemotherapy; NA: not available; * significant difference.

## Discussion

In this meta-analysis, we provided evidence that, in patients with advanced NSCLC, the variant XRCC3 241Met allele could predict good response to platinum-based chemotherapy, especially in Caucasian population; while there was no significant association of XRCC3 Thr241Met polymorphism with survival. Additionally, it was reported for the first time that the XRCC3 Thr241Met polymorphism is associated with response to platinum-based chemotherapy.

It has been well documented that impaired DNA repair capacity caused by functional SNPs of DNA repair genes is associated with survival of platinum-based chemotherapy[6], [29], [30]. It was found that the level of cisplatin DNA adducts was higher in patients with longer survival [31]. Molecular epidemiology studies also found that among NSCLC patients treated with cisplatin-based chemotherapy, individuals with impaired DNA repair capacity had a longer survival [32]. The XRCC3 gene is critical DSB repair and previous studies have shown the XRCC3 Met241Met genotype is associated with higher level of bulky DNA adducts regardless of smoking status[33]. Thus, the XRCC3 Thr241Met polymorphism might be a potential biomarker to predict clinical outcomes of NSCLC patients treated with platinum-based chemotherapy and a lot of clinical studies have been conducted to evaluate the predictive value of XRCC3 Thr241Met polymorphism[11], [12], [23], [25].

The correlation between XRCC3 Thr241Met polymorphism and response to platinum-based chemotherapy has been extensively investigated[13], [17], [18], but no significant association has been reported to date. However, our meta-analysis results suggested that carriers of the variant 241Met allele were significantly associated with good response compared with those harboring the wild 241Thr allele (Met vs. Thr, OR = 1.453, 95% CI: 1.116-1.892 and ThrMet+MetMet vs. ThrThr, OR = 1.476, 95% CI: 1.087–2.004). The reason why no significant association was found in previous studies may lie in that the sample size in these studies was small and could not provide enough statistical power. Whereas, our meta-analysis can offer enough statistical power by including 1289 participants and utilizing fixed-effects model[34] (because no significant heterogeneity was observed). Additionally, the pooled ORs were still significant when estimated by random-effects model (Met vs. Thr, OR = 1.454, 95% CI: 1.117–1.893 and ThrMet+MetMet vs. ThrThr, OR = 1.468, 95% CI: 1.074–2.006), which suggests the stability of our results. Additionally, subgroup analysis revealed that the association was significant in Caucasian population but not in Asian population, which suggested the ethnic differences. However, the significant association was based on a small number of studies and may easily be influenced by bias and other factors, like chemotherapy regimens, age, and gender. Further studies are warranted to validate this finding.

For overall survival, we did not find any significant association between XRCC3 Thr241Met polymorphism and survival of NSCLC patients in overall analysis (ThrMet+MetMet vs. ThrThr, OR = 1.074, 95% CI: 0.904–1.277). In the subgroups of platinum-based chemotherapy and radiotherapy+chemotherapy, there was no significant association either. Notably, in the heterozygote comparison, we observed a significant association of ThrMet genotype with poor survival with random-effects model but not with fixed-effects model. It has been demonstrated that, compared with random-effects model, fixed-effects model increases statistical power of meta-analyses[34]. Therefore, we surmise that this may be validated in the future after more studies are published.

Published evidence showed that the XRCC3 Thr241Met polymorphism may predict survival of NSCLC patients in a chemotherapy regimen-dependent manner [11], [12]. In an observational study of 135 NSCLC patients treated with cisplatin+gemcitabine, de las Peñas and colleagues[11] found patients with the MetMet genotype (16 months) were associated with significantly longer survival compared those with ThrMet (10 months) or ThrThr (14 months) genotype. This association was confirmed by Chen X et al[12]. Additionally, Chen X found that in the sub-population of NSCLC patients receiving non-gemcitabine regimens, the survival in patients with genotype of XRCC3 ThrThr was significantly longer than those with ThrMet or MetMet. The above evidence indicated that the XRCC3 Thr241Met polymorphism might play a role in the pharmacology of gemcitabine. Limited by the number of studies, stratified analysis by chemotherapy regiments was impossible.

Despite the effort to perform a comprehensive meta-analysis, limitations of our meta-analysis should be noted. For analysis of overall survival, we included patients with surgery or radiotherapy in addition to platinum-based chemotherapy, which may introduce heterogeneity among studies. As shown in Table 3, heterogeneity was acceptable in most comparisons and significant heterogeneity was only found in homozygote comparison. Second, limited by the number of eligible studies and no access to individual data, we did not perform stratified analysis by chemotherapy regimens. Third, safety is a major concern of the management of platinum-based chemotherapy; however, we did not assess the predictive value of XRCC3 Thr241Met polymorphism on toxicity profiles. In addition, few studies have investigated the relationship between XRCC3 Thr241Met polymorphism and toxicities, and further studies are needed.

To summary, results from our meta-analysis show that the XRCC3 Thr241Met polymorphism can predict good response to platinum-based chemotherapy in patients with advanced NSCLC, especially in Caucasian population; while there is no significant association of XRCC3 Thr241Met polymorphism with survival of NSCLC. Well-designed studies with large sample size are warranted to validate our findings and evaluate the predictive value of XRCC3 Thr241Met polymorphism on toxicity.

## Supporting Information

Figure S1 **Funnel plot of the comparison of ThrMet+MetMet vs. ThrThr for response to platinum-based chemotherapy.** The each circle represent one included study and its weight. p = 1 for Begg's test and p = 0.934 for Egger's test.
(TIF)

Figure S2 **Forest plot of the comparison of ThrMet vs. ThrThr for overall survival estimated by random-effects model.** HR = 1.220, 95% CI: 0.957–1.555, p = 0.056 for heterogeneity.
(TIF)

Figure S3 **Forest plot of the comparison of ThrMet vs. ThrThr for overall survival estimated by fixed-effects model.** HR = 1.357, 95% CI: 1.211–1.521, p = 0.056 for heterogeneity.
(TIF)

Checklist S1 **PRISMA Checklists.**
(DOC)
