# Supplementary figures and images for: XRCC3 Thr241Met Is Associated with Response to Platinum-Based Chemotherapy but Not Survival in Advanced Non-Small Cell Lung Cancer
Source: PLoS One. 2013 Oct 8;8(10):e77005. doi: 10.1371/journal.pone.0077005 (PMC3792919; doi:10.1371/journal.pone.0077005)

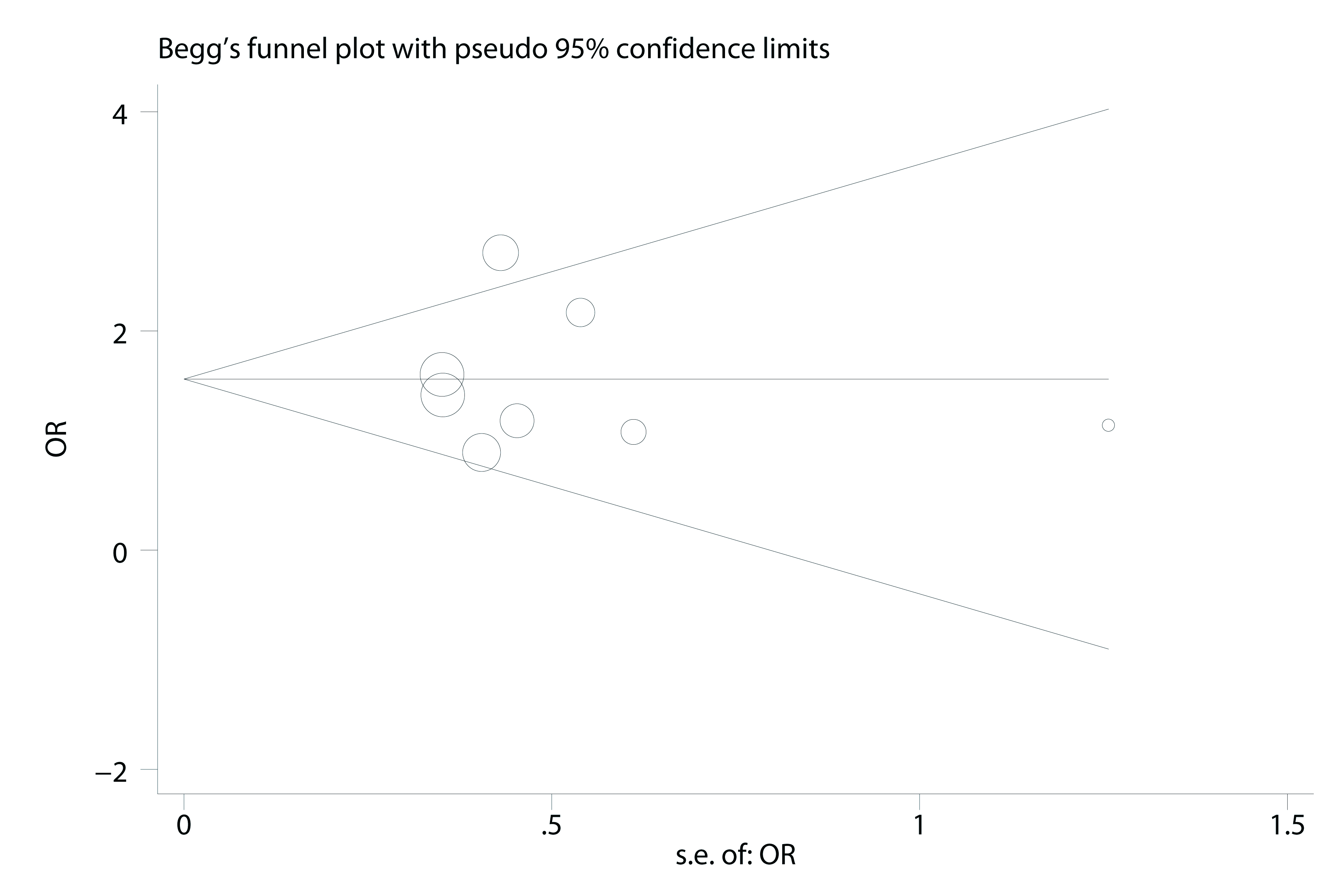

Supplement: Figure S1 — Funnel plot of the comparison of ThrMet+MetMet vs. ThrThr for response to platinum-based chemotherapy. The each circle represent one included study and its weight. p = 1 for Begg's test and p = 0.934 for Egger's test. (TIF) [file pone.0077005.s001.tif]

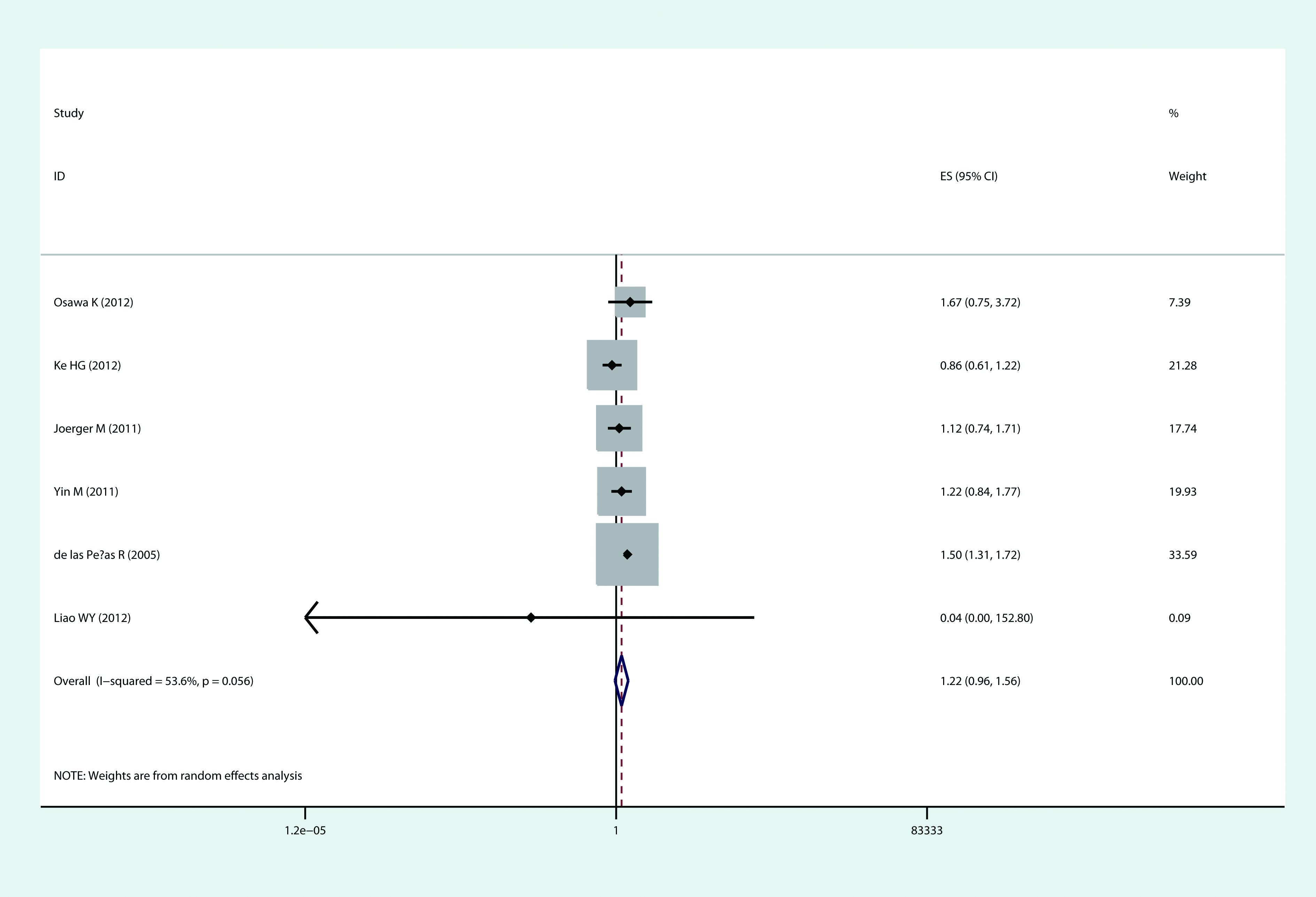

Supplement: Figure S2 — Forest plot of the comparison of ThrMet vs. ThrThr for overall survival estimated by random-effects model. HR = 1.220, 95% CI: 0.957–1.555, p = 0.056 for heterogeneity. (TIF) [file pone.0077005.s002.tif]

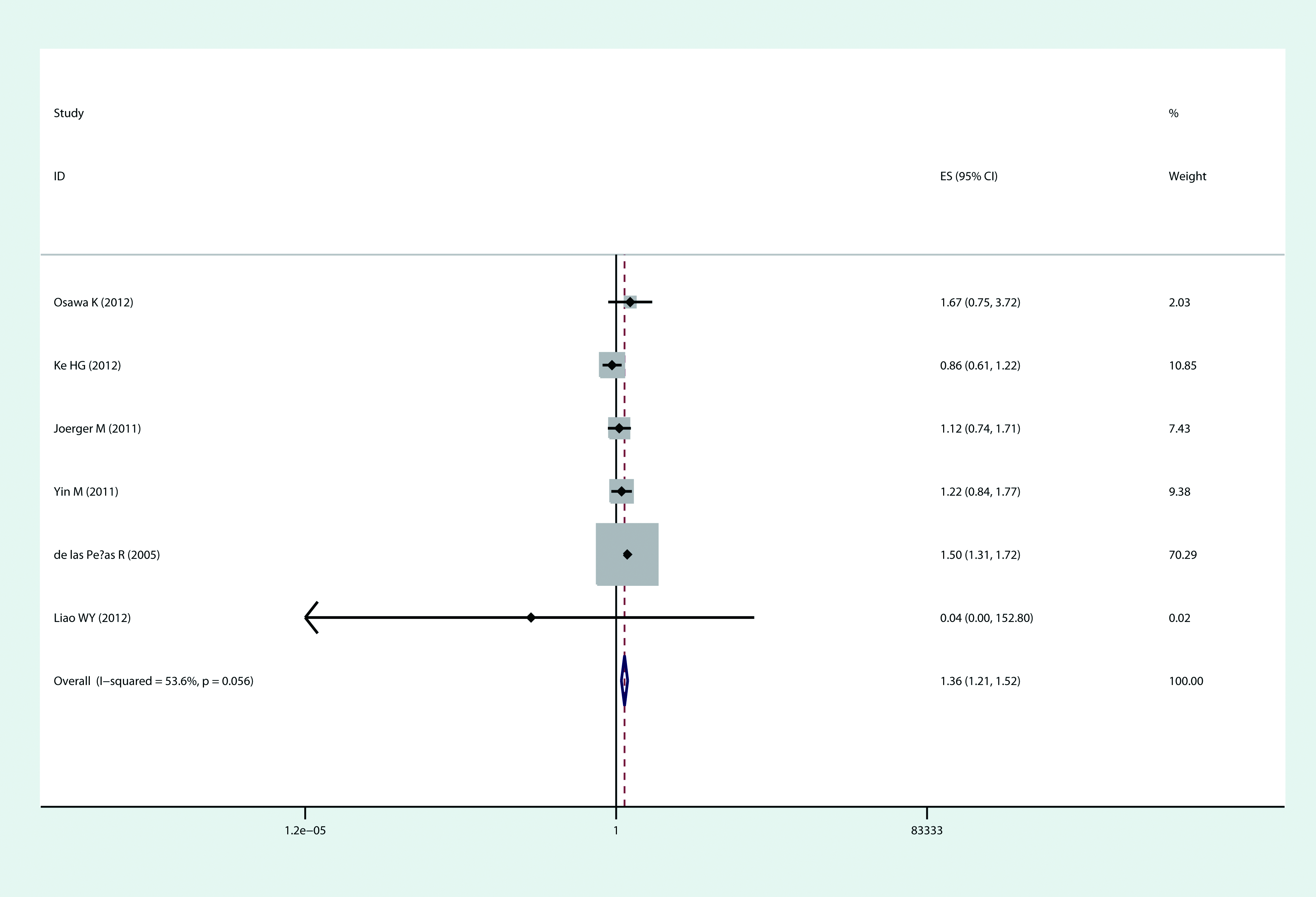

Supplement: Figure S3 — Forest plot of the comparison of ThrMet vs. ThrThr for overall survival estimated by fixed-effects model. HR = 1.357, 95% CI: 1.211–1.521, p = 0.056 for heterogeneity. (TIF) [file pone.0077005.s003.tif]
